# Macrophage-derived extracellular vesicles in the remodeling of the prostate cancer immune microenvironment and therapeutic resistance

**DOI:** 10.1186/s12967-026-08074-5

**Published:** 2026-04-04

**Authors:** Zixiong Chai, Xingyuan Dong, Songzhou Li, Menghuan Dai, Haolin Li, Zhiwei He, Peng Gu

**Affiliations:** 1 Department of Urology, The First Affiliated Hospital of Kunming Medical University, Kunming, Yunnan China; 2 Honghe Health Vocational College, Honghe, Yunnan China; 3 Yunnan Clinical Research Center for Chronic Kidney Disease, Kunming, China

**Keywords:** Prostate cancer, Tumor-associated macrophage, Extracellular vesicle, Immune microenvironment, Therapeutic resistance

## Abstract

**Background:**

Prostate cancer (PCa) is a biologically heterogeneous malignancy of the male genitourinary tract. Once the disease progresses to advanced stages, particularly castration-resistant PCa (CRPC), available treatment options become significantly limited. Although immunotherapy has demonstrated substantial clinical success in various solid tumors, its clinical benefit in PCa has been largely disappointing, mainly due to the profoundly immunosuppressive tumor microenvironment (TME).

**Main body:**

Tumor-associated macrophages (TAMs) represent a dominant immune cell population within the PCa immune microenvironment, and their functional states are closely linked to tumor progression and therapeutic responsiveness. Emerging evidence indicates that TAMs actively communicate with tumor cells and other immune subsets through the secretion of extracellular vesicles (EVs). These vesicles serve as important mediators of intercellular signaling, contributing to immune suppression, tumor progression, and the development of resistance to therapy. In this review, we comprehensively summarize recent advances in understanding the biological roles of macrophage-derived EVs (Mφ-EVs) in PCa, with particular emphasis on their involvement in immune microenvironment remodeling, tumor-promoting activities, and therapeutic resistance mechanisms.

**Conclusion:**

Mφ-EVs have emerged as key regulators of immunosuppression and treatment failure in PCa. A deeper understanding of their functional networks may provide novel opportunities for the development of EV-based diagnostic biomarkers and therapeutic strategies, ultimately helping to optimize immunotherapy and improve clinical outcomes in PCa.

**Graphical Abstract:**

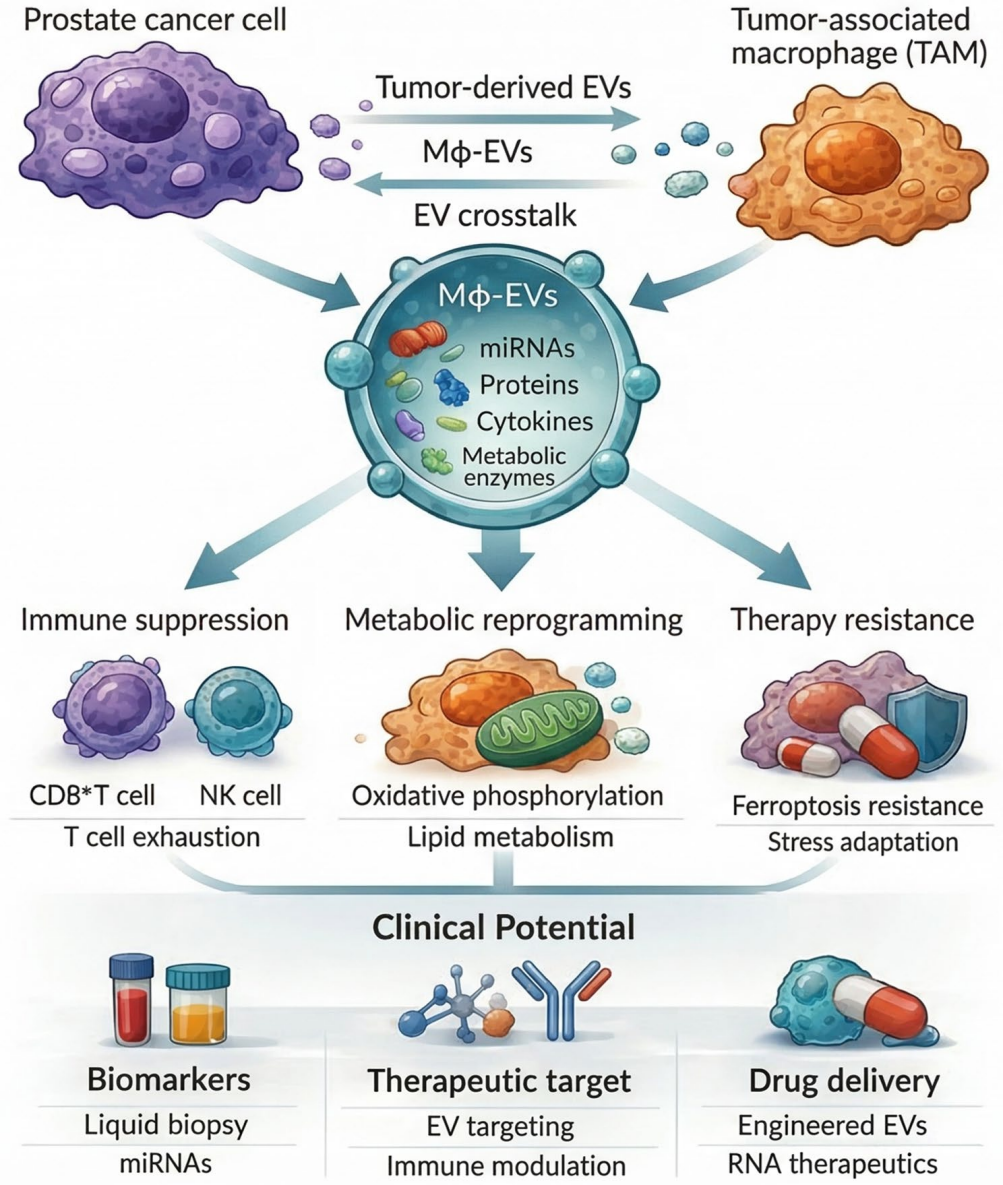

## Introduction

Prostate cancer (PCa) is among the most common malignancies affecting the male genitourinary system and is characterized by significant biological heterogeneity [1, 2]. Patients diagnosed with localized disease frequently experience favorable outcomes following radical prostatectomy, radiotherapy, or androgen deprivation therapy (ADT). However, individuals with metastatic disease or progression to metastatic castration-resistant PCa (mCRPC) continue to experience substantial therapeutic challenges and unfavorable clinical outcomes [3]. In recent years, the introduction of next-generation androgen receptor–targeted agents, cytotoxic chemotherapy, and radioligand therapy has modestly improved survival among advanced PCa patients. Nevertheless, disease progression and the emergence of therapeutic resistance remain largely inevitable. Although immunotherapy, particularly immune checkpoint inhibitors (ICIs), has transformed the treatment landscape for several solid tumors, including melanoma and non-small cell lung cancer (NSCLC), its clinical efficacy in PCa has been largely disappointing [4–8]. This limited efficacy is generally attributed to the profoundly immunosuppressive tumor microenvironment (TME) characteristic of PCa.

Several biological features of PCa contribute to establishing an immunosuppressive TME, thereby limiting the effectiveness of immune checkpoint blockade therapies. Compared to melanoma or NSCLC, PCa typically possesses a relatively low tumor mutational burden (TMB) and reduced neoantigen load, impairing effective T-cell priming and immune recognition [9, 10]. Additionally, the PCa microenvironment contains numerous immunosuppressive cell types, including tumor-associated macrophages (TAMs), regulatory T cells (Tregs), and myeloid-derived suppressor cells (MDSCs). These cells secrete inhibitory cytokines such as interleukin (IL)-10 and transforming growth factor-β (TGF-β) and express immune checkpoint molecules, collectively inhibiting cytotoxic T-cell activity. Moreover, impaired antigen presentation and limited T-cell infiltration result in the “immune-cold” phenotype commonly associated with PCa [11–13]. Among these immunosuppressive cells, TAMs have emerged as dominant and functionally diverse components of the PCa microenvironment.

Within the PCa immune microenvironment, macrophages constitute one of the most abundant and functionally critical immune subsets. TAMs often infiltrate tumor tissues, predominantly exhibiting an immunosuppressive, tumor-promoting phenotype. Their functional states and secretory profiles significantly influence immune evasion, tumor progression, and therapeutic resistance. Importantly, cells within the TME do not function in isolation; rather, they engage in highly dynamic and complex intercellular communication networks. Beyond soluble cytokines, chemokines, and direct cellular contacts, extracellular vesicle (EV)-mediated communication is emerging as a pivotal mechanism for maintaining TME homeostasis and coordinating immune regulation [14–16].

EVs are nanoscale, membrane-bound vesicles actively released by cells and include subtypes, such as exosomes, microvesicles, and apoptotic bodies. These vesicles carry various bioactive cargos, including proteins, lipids, and nucleic acids, thereby facilitating precise modulation of immune responses and TME remodeling [17–20]. Increasing evidence suggests that EVs are crucial mediators of intercellular communication and functional regulation within the TME, broadly participating in immune modulation, tumor progression, and therapy resistance.

Among diverse cellular sources of EVs in the TME, macrophage-derived EVs (Mφ-EVs) have recently drawn attention due to their strong immunomodulatory potential. Notably, Mφ-EVs function as potent immunoregulatory messengers, serving as vital links between tumor cells and diverse immune cell populations. Through these mechanisms, they exert multiple effects on sustaining immunosuppression, promoting tumor progression, and facilitating therapeutic resistance [21].

Importantly, Mφ-EVs possess a unique repertoire of bioactive molecules reflecting the activation state of their parent macrophages. These vesicles harbor various functional cargos, including immunoregulatory cytokines, metabolic enzymes, signaling proteins, and regulatory non-coding RNAs such as microRNAs (miRNAs) and long non-coding RNAs (lncRNAs) [22, 23]. Through cargo delivery, Mφ-EVs regulate multiple biological processes in recipient cells, including immune cell activation, inflammatory signaling, metabolic reprogramming, and cellular stress responses [24, 25].

Accordingly, systematic elucidation of the mechanisms by which Mφ-EVs regulate the PCa immune microenvironment, as well as their roles in therapeutic response and resistance, is of considerable importance. Such insights will deepen our understanding of immune escape in PCa and may facilitate the identification of novel diagnostic biomarkers and therapeutic targets, ultimately contributing to the optimization of immunotherapeutic strategies in clinical practice.

## Macrophages and Mφ-EVs

### Biological characteristics and functional diversity of macrophages

Macrophages are central effector cells of the innate immune system, distributed widely across almost all tissues. They are essential for maintaining tissue homeostasis, orchestrating immune defense, and regulating inflammatory responses. Core biological functions of macrophages include the phagocytic clearance of pathogens and cellular debris, antigen uptake and presentation, and the secretion of diverse cytokines and chemokines. Thus, they function as critical mediators between innate and adaptive immunity [26, 27]. A defining feature that distinguishes macrophages from many other immune cell types is their remarkable plasticity and capacity to adapt to various microenvironmental cues [28].

In response to distinct environmental signals, macrophages undergo dynamic phenotypic and functional changes. Within the classical paradigm, macrophage activation is generally categorized into classically activated (M1-like) and alternatively activated (M2-like) states. M1-like macrophages are typically induced by interferon-γ (IFN-γ) and lipopolysaccharide (LPS) and possess proinflammatory and antitumor properties. These cells directly promote tumor cell death through the production of tumor necrosis factor-α (TNF-α), IL-12, nitric oxide, and other bioactive mediators, while simultaneously enhancing T cell-mediated antitumor immune responses [29–33]. In contrast, cytokines such as IL-4, IL-10, and IL-13 predominantly drive M2-like macrophage polarization. These macrophages exert immunosuppressive functions and contribute to tissue remodeling and angiogenesis. They achieve these effects primarily through secreting IL-10, TGF-β, and vascular endothelial growth factor (VEGF), thus promoting tumor growth and metastasis [34–39].

**Fig. 1:**
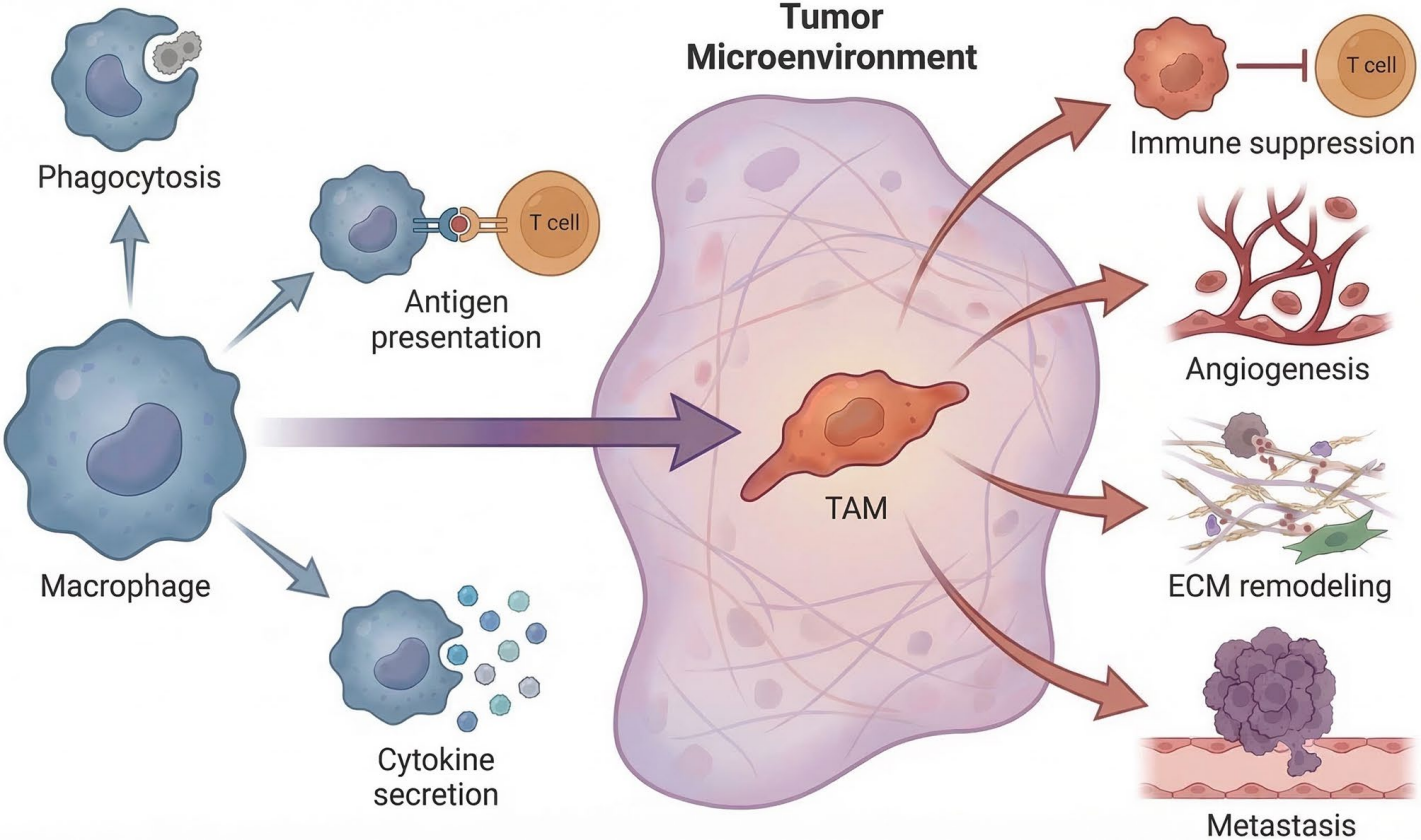
Biological functions and reprogramming of macrophages into TAMs in the TME. Under homeostatic conditions, macrophages perform key innate immune functions, including phagocytosis, antigen presentation, and cytokine secretion. Within the TME, diverse signals reprogram macrophages into TAMs that predominantly exhibit immunosuppressive (M2-like) features and promote tumor progression via immune suppression, angiogenesis, ECM remodeling, and facilitation of metastasis

Within the TME, macrophages are commonly reprogrammed into TAMs (Fig. 1). Increasing evidence indicates that TAMs do not strictly adhere to the binary M1/M2 classification but instead occupy a continuum of activation states, predominantly featuring M2-like characteristics [40–43]. In PCa and other solid malignancies, TAMs are often highly enriched and promote tumor progression through multiple mechanisms, including suppression of effector T-cell activity, enhancement of tumor angiogenesis, remodeling of the extracellular matrix (ECM), and facilitation of tumor cell invasion and metastasis [44–48]. Clinical studies have further demonstrated that increased TAM infiltration correlates with unfavorable prognosis, disease progression, and therapeutic resistance in PCa, underscoring their relevance as key cellular targets in tumor immunotherapy [49, 50].

### Biological characteristics and secretion of EVs

EVs represent a heterogeneous population of membrane-bound vesicles released by cells through endosomal pathways or direct outward budding of the plasma membrane. Based on their biogenesis and size distribution, EVs are generally classified into exosomes (~40–160 nm in diameter), microvesicles (~100–1000 nm), and apoptotic bodies (>1000 nm) [51, 52]. Among these subtypes, exosomes have been extensively studied and are commonly regarded as functionally representative EVs in tumor biology.

The biogenesis of EVs is tightly regulated by intracellular trafficking processes. Exosomes originate from the endosomal system and are released into the extracellular space following the fusion of multivesicular bodies (MVBs) with the plasma membrane, whereas microvesicles are generated through direct outward budding of the plasma membrane [53–55]. The lipid bilayer structure of EVs confers protection to their molecular cargos against degradation by extracellular nucleases and proteases, thereby enabling stable and long-range intercellular communication [56–58].

EV cargos encompass a wide array of bioactive molecules, including proteins, lipids, DNA, and diverse classes of non-coding RNAs, such as miRNAs, lncRNAs, and circular RNAs (circRNAs) [59, 60]. Notably, the molecular composition of EVs largely reflects the biological state and functional characteristics of their cells of origin.

**Fig. 2:**
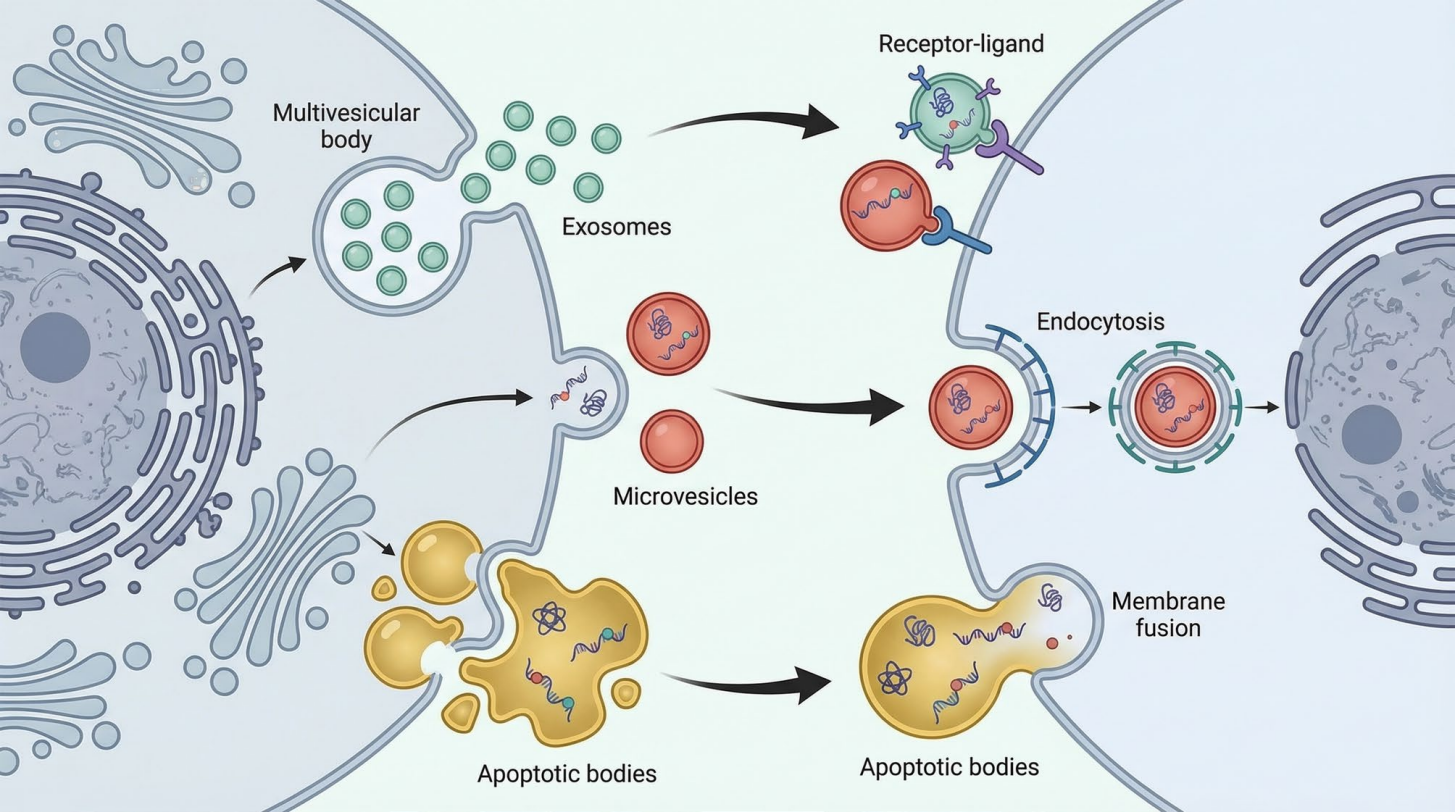
Biogenesis and intercellular communication mechanisms of EVs

EVs interact with recipient cells through multiple mechanisms, including receptor-ligand interactions, endocytosis, and direct membrane fusion, thereby delivering their cargos into target cells and modulating downstream cellular functions (Fig. 2) [61]. Within the TME, EVs are increasingly recognized as critical mediators of communication between tumor cells and immune cells, broadly participating in tumor cell proliferation, invasion, metastasis, immune evasion, and the development of therapeutic resistance.

Schematic illustration of EV biogenesis and uptake pathways. Left: major EV subtypes and their origins (exosomes from MVBs, microvesicles budding from the plasma membrane, apoptotic bodies from cell fragmentation). Right: principal mechanisms of EV interaction with recipient cells (receptor-ligand binding, endocytosis, membrane fusion), enabling delivery of protein, lipid, and nucleic acid cargos

Importantly, EVs exhibit remarkable stability in biological fluids, and their cargos often harbor tumor-specific molecular signatures, rendering them attractive candidates for liquid biopsy (LB)-based biomarkers [62–64]. Concurrently, advances in EV engineering technologies have highlighted their potential as versatile platforms for drug delivery and immune modulation [65–67]. Collectively, these features position EVs as promising tools in precision oncology and translational cancer research.

## Mechanistic roles of Mφ-EVs in the tumor immune microenvironment

### Regulation of macrophage polarization and immune homeostasis by EVs

Within the tumor immune microenvironment, EVs are not only derived from tumor cells; infiltrating immune cells, particularly macrophages, also represent a major source of EVs [68, 69]. Growing evidence indicates that the biological functions of Mφ-EVs depend significantly on the activation state of their parental macrophages [70, 71]. EVs released from macrophages with distinct phenotypic programs differ markedly in their molecular composition and functional outputs. Consequently, they exert finely tuned, amplified regulatory effects in the TME [70, 72–74].

In general, EVs derived from M1-like macrophages are enriched in proinflammatory miRNAs, such as miR-155 and miR-125b, as well as immune-activating proteins. These vesicles enhance antigen presentation and promote effector T-cell activation, thus reinforcing antitumor immune responses [72, 75–77]. Conversely, EVs released from M2-like or TAM-like macrophages preferentially carry immunosuppressive cargos, including miR-223, miR-146a, TGF-β, and arginase 1 (ARG1). Such EVs inhibit effector T-cell functions, promote Treg expansion, and reprogram metabolic and inflammatory signaling pathways, thereby maintaining an immunosuppressive TME [72, 78]. Consistent with these observations, multiple studies have demonstrated that EVs derived from M1-like macrophages inhibit tumor cell proliferation and invasion, while EVs from M2-like macrophages promote tumor growth, metastasis, and therapeutic resistance [24, 73, 79–82]. This phenomenon, often described as a “functional mirroring” effect, underscores the decisive influence of macrophage phenotype on the biological activities of their secreted EVs.

The close alignment between macrophage activation states, EV cargo composition, and downstream functional outcomes positions Mφ-EVs as critical amplifiers of signals in tumor immune regulation. These vesicles act as extracellular extensions of macrophage functional programs, disseminating immunostimulatory or immunosuppressive signals throughout the TME. Moreover, their miRNA and protein cargos induce persistent functional changes in recipient cells via canonical pathways, including STAT3, PI3K-AKT, and NF-κB [47, 83–85].

Notably, although these mechanisms are conserved across multiple solid tumor types, EV-mediated bidirectional communication between tumor cells and macrophages appears particularly pronounced in PCa, a malignancy characterized by a profoundly immunosuppressive immune landscape. In this context, macrophage-tumor crosstalk via EVs plays a critical role in driving TAM polarization, maintaining immunosuppressive homeostasis, and facilitating disease progression, exhibiting distinct tumor-type specificity [86]. The following sections summarize recent advances elucidating these processes in PCa. Table 1 provides a summary of Mφ-EV cargos, target cell types, functional effects, and key signaling mechanisms in the PCa immune microenvironment.

**Table 1:** Functions of Mφ-EVs in the PCa immune microenvironment

Target cell/type	Mφ-EVs Cargo	Functional Effects/Biological Roles	Key Signaling Pathways/ Mechanisms
Macrophages (self)	M1-type: miR-155, miR-125b M2/TAM-type: miR-223, miR-146a, TGF-β, ARG1	M1-EVs: enhance antigen presentation, activate effector T cells M2-EVs: suppress effector T cells, promote Treg expansion, maintain immunosuppressive environment	STAT3, PI3K-AKT, NF-κB
Effector T cells (CD8^+^/CD4^+^)	PD-L1, ARG1, miRNAs	Inhibit activation and cytotoxicity, induce exhaustion	Direct inhibitory signaling
Tregs	TGF-β, IL-10	Promote differentiation and expansion, reinforce immunosuppression	Immunoregulatory signaling
NK cells	miR-21, miR-29a	Reduce degranulation and cytotoxic molecule expression, impair killing function	Immune evasion
Tumor cells	Antioxidant proteins (e.g., PRDX6), metabolic enzymes, regulatory miRNAs	Increase tolerance to therapeutic stress, support metabolic adaptation, promote therapy resistance	Antioxidant defense, lipid peroxidation prevention, metabolic reprogramming
CAFs	miRNAs, proteins, TGF-β	Induce tumor-promoting phenotype, enhance ECM deposition, promote immune exclusion	Intercellular communication, microenvironment remodeling
Tumor-macrophage feedback loop	TAM-EVs and tumor-derived EVs	Amplify M2/TAM polarization, stabilize immunosuppressive environment	Bidirectional EV-mediated communication

### EV-mediated immunosuppression and immune evasion

Beyond influencing macrophage polarization, Mφ-EVs exert profound immunomodulatory effects by reprogramming the functions of other immune cell populations, thereby directly facilitating tumor immune evasion. Through delivering miRNAs, proteins, and lipid mediators, these vesicles disrupt immune surveillance networks and attenuate the host’s capacity to recognize and eliminate malignant cells.

Accumulating evidence demonstrates that EVs released by TAMs (TAM-EVs) suppress antitumor immunity by transferring immunoregulatory non-coding RNAs, including miR-21 and miR-29a, which downregulate signaling pathways essential for effector T-cell activation. Consequently, CD8^+^ T-cell proliferation and cytotoxic functions are significantly impaired. Additionally, TAM-EVs interfere with the antitumor activity of natural killer (NK) cells by reducing their degranulation capacity and the expression of cytotoxic effector molecules [69, 87, 88]. Through this multitargeted, low-intensity yet sustained mode of regulation, TAM-EVs progressively establish an immunosuppressive milieu within the TME, thereby facilitating immune escape.

Importantly, a complex feedback regulatory network exists between tumor cell-derived EVs and Mφ-EVs. Tumor-derived EVs can drive macrophage polarization toward immunosuppressive phenotypes, while the resulting TAMs, in turn, release EVs that further amplify immunosuppressive signaling. This EV-mediated tumor-macrophage communication axis forms a self-reinforcing positive feedback loop that stabilizes immune suppression within the TME [89, 90]. In PCa, this bidirectional EV-driven crosstalk is increasingly recognized as a critical mechanism underlying persistent immune tolerance and resistance to immunotherapy.

### Mediation of therapeutic resistance and cellular stress responses

Emerging evidence indicates that Mφ-EVs can directly enhance tumor cell tolerance to therapeutic stress by transferring antioxidant-associated proteins. For example, EV-mediated delivery of peroxiredoxin 6 (PRDX6) has been shown to increase resistance to lipid peroxidation, thereby suppressing lipid peroxidation-dependent forms of cell death, including ferroptosis [91–93]. These findings highlight a previously underappreciated mechanism by which the immune microenvironment contributes to tumor metabolic adaptation and stress resilience through EV-dependent signaling, ultimately facilitating the development of therapeutic resistance.

In PCa, escalating therapeutic pressure is accompanied by dynamic alterations in both the abundance and functional states of TAMs. TAM-secreted EVs may enable tumor cells to adapt to stress conditions induced by ADT, chemotherapy, or radiotherapy, thus promoting the emergence of castration-resistance and multidrug-resistant phenotypes [94–96]. This EV-mediated macrophage-tumor interaction provides an immune microenvironment-centered framework for understanding treatment failure in advanced PCa.

Beyond enhancing tumor resistance to therapeutic stress, Mφ-EV-mediated communication is increasingly recognized as a regulator of metabolic reprogramming in the PCa microenvironment. Tumor progression under therapeutic stress frequently involves profound metabolic changes in cancer cells and immune populations, including TAMs. Evidence indicates that Mφ-EVs transfer metabolic enzymes, regulatory miRNAs, and signaling molecules, altering cellular metabolic pathways in recipient tumor and immune cells [97–100]. Macrophage metabolic pathways strongly influence their polarization: proinflammatory macrophages predominantly rely on glycolysis, whereas immunosuppressive TAMs mainly utilize oxidative phosphorylation and fatty acid metabolism [101–103]. Thus, the exchange of metabolic regulators through Mφ-EVs between TAMs and tumor cells reinforces tumor-promoting metabolic states within the microenvironment. This further maintains immunosuppression and facilitates therapeutic resistance. Collectively, these findings suggest that Mφ-EV-mediated metabolic crosstalk represents another regulatory mechanism connecting TAM functions with treatment resistance in advanced PCa.

### EV-mediated intercellular communication with fibroblasts and T cell subsets

Beyond their direct effects on tumor cells, TAMs engage in extensive intercellular communication with non-malignant stromal and immune cell populations through the secretion of EVs, thereby reshaping the TME at a systems level. Notably, TAM-derived EVs can be internalized by cancer-associated fibroblasts (CAFs), where the delivery of miRNAs, proteins, and TGF-β-related signaling components induces a tumor-promoting fibroblast phenotype. This reprogramming enhances ECM deposition and promotes immune exclusion, indirectly suppressing antitumor immune responses [104–106].

In parallel, TAM-derived EVs exert direct regulatory effects on T cell populations. On the one hand, immunosuppressive cargos carried by these vesicles, such as programmed death ligand 1 (PD-L1), ARG1, and specific miRNAs, impair the activation and cytotoxic functions of CD8^+^ and CD4^+^ effector T cells, thereby promoting T cell dysfunction and exhaustion. On the other hand, EVs enriched in TGF-β and IL-10 facilitate the differentiation and expansion of Tregs, further reinforcing local immunosuppression [86, 107–109].

Collectively, TAM-derived EVs orchestrate a multilayered immunosuppressive network within the PCa immune microenvironment by coordinately regulating fibroblasts and diverse T cell subsets. This EV-centered communication axis likely represents a critical barrier limiting the efficacy of current immunotherapeutic strategies and a key driver of tumor progression.

## Recent advances in Mφ-EVs in PCa

### Remodeling of the immune microenvironment through the tumor–macrophage EV axis

Within the PCa immune microenvironment, tumor cells and immune cells establish a highly dynamic and complex communication network mediated by EVs. Among these interactions, tumor cell-derived EVs play a particularly critical role in shaping macrophage polarization states. Accumulating evidence demonstrates that EVs secreted by PCa cells can be efficiently internalized by infiltrating monocytes or unpolarized (M0) macrophages, thereby driving their differentiation toward an immunosuppressive, TAM-like phenotype [110, 111].

Experimental studies using the classical PCa cell line PC3 have shown that PC3-derived EVs robustly induce macrophage polarization toward an immunosuppressive TAM-like state. Specifically, EVs released from PC3 cells markedly promote the acquisition of M2/TAM-associated cytokine profiles in M0 macrophages differentiated from the human monocytic leukemia cell line THP-1. This phenotypic shift is characterized by a significant increase in the secretion of the anti-inflammatory cytokine IL-10 and a concomitant reduction in the expression of the proinflammatory cytokine IL-12 [112]. Such EV-mediated macrophage reprogramming not only attenuates local antitumor immune responses but also fosters a tumor-supportive microenvironment by enhancing the production of multiple protumorigenic factors. Consistently, in vitro functional assays have demonstrated that macrophages polarized by PC3-derived EVs significantly enhance PCa cell proliferation and migratory capacity, underscoring the central role of tumor EV-induced macrophage reprogramming in immune microenvironment remodeling [47, 112].

At the molecular level, PCa-derived EVs exert their regulatory effects on macrophage function through specific cargo molecules, including miRNAs and functional proteins. Notably, EVs released by tumor cells under hypoxic conditions have been shown to be enriched in miR-301a-3p, which promotes macrophage polarization toward an immunosuppressive M2-like phenotype by targeting phosphatase and tensin homolog (PTEN) and activating the phosphoinositide 3-kinase γ (PI3Kγ) signaling pathway. Although this mechanism was initially characterized in pancreatic cancer, accumulating evidence suggests that a similar EV-mediated regulatory paradigm may operate in PCa [113–115]. Macrophages polarized through this pathway exhibit elevated expression of M2-associated markers, such as CD206 and Arg1, along with enhanced secretion of anti-inflammatory mediators, leading to profound remodeling of their immunophenotype and secretory profile. Ultimately, this EV-driven macrophage reprogramming establishes an immune microenvironment conducive to tumor survival and progression, significantly enhancing PCa cell migration, invasion, and epithelial-mesenchymal transition (EMT) [90, 116].

These findings indicate that PCa cells are not passive recipients of immune regulation but actively educate macrophages through EV secretion, converting them into key facilitators of tumor progression. The EV-mediated positive feedback loop between tumor cells and macrophages likely represents a central mechanism underlying PCa progression and limited immunotherapy efficacy, providing a rationale for therapeutic targeting of the TAM-EV axis.

### Impact of Mφ-EVs on therapeutic response and resistance

Beyond remodeling the immune microenvironment, Mφ-EVs are increasingly recognized as critical regulators of therapeutic responses and resistance. TAMs and their secreted EVs can modulate tumor cell survival signaling and stress-response pathways through the transfer of functional cargos, including miRNAs and proteins, thereby altering tumor sensitivity to conventional treatments such as chemotherapy and radiotherapy [117]. For instance, EVs derived from M2-polarized macrophages promote resistance to multiple anticancer agents, including paclitaxel, cisplatin, and gemcitabine. Mechanistically, these EVs enhance tumor cell survival by activating the PI3K/AKT signaling pathway, suppressing apoptotic cascades, or regulating the expression of genes involved in drug metabolism and detoxification, ultimately reducing the cytotoxic efficacy of chemotherapeutic agents [73, 118, 119]. Although current evidence primarily comes from in vitro studies and tumor models other than PCa, these findings collectively suggest that EV-mediated resistance mechanisms driven by the immune microenvironment may represent a broadly conserved phenomenon. This perspective provides new insights into the mechanisms underlying therapeutic failure in PCa.

Additionally, tumor cell-derived EVs have also been reported to carry immune checkpoint molecules such as PD-L1. Emerging evidence indicates that EV-associated PD-L1 can function as a “molecular decoy” by sequestering therapeutic antibodies, thereby attenuating the efficacy of immune checkpoint blockade therapies [120]. While this mechanism has been experimentally validated in several solid tumors, including NSCLC, its potential relevance in PCa warrants further investigation and may offer important implications for optimizing immunotherapeutic strategies.

### Potential clinical applications of Mφ-EVs in PCa

EVs exhibit remarkable stability in biological fluids and can partially recapitulate the molecular characteristics of their cells of origin, rendering them attractive candidates for LB and precision medicine applications. Substantial numbers of EVs are detectable in plasma, urine, and other body fluids of PCa patients, carrying diverse bioactive cargos, such as proteins, miRNAs, and other non-coding RNAs, that display tumor-associated molecular signatures [121–123].

Most studies investigating the diagnostic and prognostic value of EVs in PCa have focused on heterogeneous populations of circulating EVs. In contrast, analyses specifically targeting immune cell-derived EVs, particularly those originating from macrophages, remain relatively limited. Current approaches generally involve isolating EVs from patient plasma or urine followed by molecular profiling of their cargos, such as miRNAs or proteins, as potential biomarkers for early detection, disease stratification, prognostic evaluation, and therapeutic monitoring. Accumulating evidence suggests that specific EV-associated miRNAs, including miR-375 and miR-1290, are differentially expressed between localized and metastatic PCa and correlate with tumor grade, aggressiveness, prostate-specific antigen (PSA) levels, disease progression risk, and patient outcomes [106, 124–129] In addition to miRNAs, several EV-associated proteins have also demonstrated potential diagnostic and prognostic value in PCa [106, 130].

Although the precise cellular origins of most circulating EVs remain unclear, the abundant infiltration of TAMs within the PCa immune microenvironment suggests that TAM-derived EVs may substantially contribute to shaping the circulating EV landscape and reflecting tumor immune status. Therefore, systematic characterization of Mφ-EV molecular signaturesand clarification of their relationships with global circulating EV profiles, may produce biologically relevant biomarkers for immune states assessment and patient risk stratification.

From a therapeutic perspective, Mφ-EVs also represent promising intervention targets. TAMs and their EVs actively sustain immunosuppressive microenvironments and promote therapeutic resistance, highlighting the TAM–EV axis as a potential target for immune modulation. Additionally, advances in EV engineering and targeted modification have positioned EVs as natural nanocarriers for the precise delivery of therapeutic agents, including anticancer drugs and nucleic acid–based therapeutics, thereby offering innovative strategies to reprogram tumor immunity [73, 131–134].

Building on these advances, hybrid nanoparticle systems have further expanded the therapeutic potential of EV-based delivery platforms. Such hybrid nanoparticles are typically generated by integrating naturally derived EV membranes, including those from Mφ-EVs, with synthetic nanomaterials such as liposomes or polymeric nanoparticles [135–137]. This strategy combines the biological advantages of EVs, including intrinsic biocompatibility, low immunogenicity, and cell-targeting capability, with the structural stability and scalable manufacturability of synthetic nanocarriers, thereby helping to overcome key barriers to the clinical translation of EV-based therapeutics. For example, EV-liposome hybrid vesicles have shown improved drug-loading efficiency and circulation stability while preserving membrane proteins required for cellular uptake [138–141]. In preclinical cancer models, these engineered systems have enhanced the delivery of chemotherapeutic agents, small interfering RNAs (siRNAs), and immunomodulatory molecules to tumor tissues [142, 143]. Although many of these studies have not specifically focused on Mφ-EVs or PCa, they provide a valuable proof of concept for the development of Mφ-EV-based delivery systems aimed at targeted drug delivery and immune modulation in PCa and other malignancies.

Although most current studies have focused on bulk EV populations or tumor cell-derived EVs, these findings provide a robust conceptual foundation for the development of immunotherapeutic approaches specifically targeting or exploiting Mφ-EVs. Overall, therapeutic strategies centered on Mφ-EVs show significant potential for improving immune-based treatments and clinical outcomes in PCa.

### Clinical studies and translational investigations of EV-based biomarkers

Recent years have witnessed increasing efforts to translate EV-based biomarkers into clinical applications. Numerous clinical studies have evaluated circulating EV-associated molecules as diagnostic and prognostic biomarkers in oncology. Plasma-derived EV miRNAs and proteins have been assessed for early detection and disease monitoring in malignancies such as PCa, pancreatic cancer, and glioblastoma [144–147]. These translational efforts contributed to the development of the ExoDx Prostate (IntelliScore) urine test, which received U.S. Food and Drug Administration (FDA) Breakthrough Device Designation. This assay detects urinary EV-associated RNA transcripts and has become a clinically available, Clinical Laboratory Improvement Amendments (CLIA)-certified EV-based test for PCa risk stratification [148–150]. Additionally, ongoing clinical studies investigate circulating EV cargos, including miRNAs and PD-L1-containing EVs, as predictive biomarkers for immunotherapy responses in various solid tumors [151–154]. Although translational and clinical investigations focusing specifically on Mφ-EVs remain limited, these efforts highlight the growing interest in EVs as minimally invasive biomarkers and therapeutic targets.

## Challenges and future perspectives

Despite growing recognition of the critical roles of Mφ-EVs in shaping the immune microenvironment of PCa, several key challenges remain. First, EV populations are inherently heterogeneous, and vesicles originating from distinct cell types within the TME are highly intermixed in vivo. This complexity poses major obstacles to the precise tracing, selective isolation, and functional attribution of TAM-derived EVs. Currently available isolation techniques-including ultracentrifugation, density gradient separation, and immunoaffinity-based approaches-remain limited in their ability to reliably discriminate EVs by cellular origin, and standardized, reproducible methodological frameworks are still lacking. Second, although existing studies have primarily focused on the immunosuppressive and therapy-resistant effects of EV-associated miRNAs and proteins, the upstream regulatory mechanisms governing TAM-EV biogenesis and cargo selection, as well as the downstream signaling networks activated in recipient cells, remain poorly defined. In particular, the dynamic patterns of TAM-derived EV secretion across different disease stages, therapeutic contexts, and spatial niches within the PCa microenvironment have yet to be systematically characterized.

These limitations are especially relevant to the use of circulating EVs as clinical biomarkers, because this application depends heavily on accurate isolation and characterization. EVs detected in blood or urine originate from multiple cellular sources, including tumor cells, immune cells, stromal cells, and normal tissues. As a result, disease-relevant signals from tumor- and TAM-associated EVs may be diluted or masked by abundant vesicles released from non-malignant cells, thereby complicating data interpretation and reducing biomarker specificity. Accordingly, more refined isolation strategies, such as immunoaffinity-based enrichment using cell type-specific surface markers, will be essential for improving the precision and clinical applicability of EV-based diagnostic assays. Such methodological advances would address a major unmet need in translating Mφ-EVs into clinically useful biomarkers for PCa. In light of these diagnostic challenges, the translational potential of TAM-derived EVs in PCa should be evaluated with careful attention to analytical specificity and cellular origin.

From a translational standpoint, the greatest potential value of TAM-derived EVs in PCa may lie in their function as immune-modulatory mediators and therapeutic targets rather than as conventional LB biomarkers alone. Developing strategies to selectively modulate the production, release, or biological activity of TAM-derived EVs in vivo, or to engineer these vesicles for therapeutic purposes, represents a critical unmet need. Integration of single-cell sequencing, multi-omics profiling, and spatial transcriptomics is expected to provide a comprehensive view of the macrophage-EV-tumor cell interaction network, thereby uncovering novel mechanistic insights and actionable targets for optimizing immunotherapy and precision interventions in PCa.

In addition to serving as modulatory targets, EVs, including Mφ-EVs, are increasingly being explored as therapeutic delivery vehicles. Compared with synthetic nanoparticles, EVs offer several inherent advantages, including biocompatibility, low immunogenicity, and the capacity to cross biological barriers. Engineered EVs have shown promise as carriers for siRNAs, chemotherapeutic agents, and immunomodulatory molecules in preclinical cancer models. However, several barriers must still be overcome before EV-based delivery systems can be broadly implemented in clinical practice, including scalable production, efficient cargo loading, targeting specificity, and regulatory standardization. Addressing these challenges will be crucial for developing Mφ-EVs as effective therapeutic platforms and for complementing immunomodulatory strategies in PCa and other malignancies.

## Conclusion

Mφ-EVs play multifaceted and essential roles in regulating the immune microenvironment, tumor progression, and therapeutic responses in PCa. As central mediators of immunosuppression and therapy resistance, understanding their biological functions and regulatory networks will enhance our knowledge of immune escape mechanisms and inform the development of innovative immune-based and precision therapies. Future efforts integrating multi-omics analyses, standardized clinical validation, and advanced EV engineering approaches are likely to establish Mφ-EVs as key drivers of tumor immune modulation and personalized treatment paradigms in PCa.

## Data Availability

The study is a review article that did not generate or analyze original data; therefore, no data are available for sharing.

## Abbreviations

ADT: Androgen deprivation therapy
AKT: Protein kinase B
ARG1: Arginase 1
CAFs: Cancer-associated fibroblasts
circRNAs: Circular RNAs
CLIA: Clinical Laboratory Improvement Amendments
CRPC: Castration-resistant PCa
ECM: Extracellular matrix
EMT: Epithelial-mesenchymal transition
EVs: Extracellular vesicles
FDA: U.S. Food and Drug Administration
ICIs: Immune checkpoint inhibitors
IFN-γ: Interferon-γ
IL: Interleukin
LB: Liquid biopsy
LPS: Lipopolysaccharide
lncRNAs: Long non-coding RNAs
M0: Unpolarized macrophages
M1: Classically activated macrophage (proinflammatory)
M1-like: Classically activated-like macrophage
M2: Alternatively activated macrophage (immunosuppressive)
M2-like: Alternatively activated-like macrophage
MDSCs: Myeloid-derived suppressor cells
miRNAs: microRNAs
Mφ-EVs: Macrophage-derived extracellular vesicles
mCRPC: Metastatic castration-resistant PCa
MVBs: Multivesicular bodies
NF-κB: Nuclear factor κ-light-chain-enhancer of activated B cells
NK: Natural killer (cell)
NSCLC: Non-small cell lung cancer
PCa: Prostate cancer
PD-L1: Programmed death-ligand 1
PI3K: Phosphoinositide 3-kinase
PRDX6: Peroxiredoxin 6
PSA: Prostate-specific antigen
PTEN: Phosphatase and tensin homolog
siRNAs: Small interfering RNAs
STAT3: Signal transducer and activator of transcription 3
TAMs: Tumor-associated macrophages
TAM-EVs: EVs released from TAMs
TGF-β: Transforming growth factor-β
TME: Tumor microenvironment
TNF-α: Tumor necrosis factor-α
TMB: Tumor mutational burden
Tregs: Regulatory T cells
VEGF: Vascular endothelial growth factor
